# Molecular characterization of CTNS mutations in Tunisian patients with ocular cystinosis

**DOI:** 10.1186/s13000-022-01221-8

**Published:** 2022-05-06

**Authors:** Latifa Chkioua, Yessine Amri, Chaima Saheli, Wassila Mili, Sameh Mabrouk, Imen Chabchoub, Hela Boudabous, Wissem Ben Azzouz, Hadhami Ben Turkia, Salima Ferchichi, Neji Tebib, Taieb Massoud, Mohamed Ghorbel, Sandrine Laradi

**Affiliations:** 1 Research Laboratory of Human Genome and Multifactorial Diseases, Faculty of Pharmacy, University of Monastir, Street Avicenne, 5000 Monastir, Tunisia; 2 Biochemistry Laboratory (LR 00SP03), Bechir Hamza Children’s Hospital, Tunis, Tunisia; 3 Ophtalmic Department, Farhat Hached Hospital, Sousse, Tunisia; 4 Pediatric Department, Sahloul Hospital, Sousse, Tunisia; 5 Pediatric Department, Hedi Chaker Hospital, Sfax, Tunisia; 6 Pediatric Department, LaRabta Hospital, Tunis, Tunisia; 7 Biochemistry Laboratory, Farhat Hached Hospital, Sousse, Tunisia; 8 The Auvergne-Rhône-Alpes Regional Branch of the French National Blood System EFS/GIMAP, EA 3064, 42100 Saint Etienne, France

**Keywords:** Ocular cystinosis, Cornea crystal, Mutations, CTNS, Tunisian families

## Abstract

**Background:**

Ocular cystinosis is a rare autosomal recessive disorder characterized by intralysosomal cystine accumulation in renal, ophthalmic (cornea, conjunctiva), and other organ abnormalities. Patients with ocular cystinosis are mostly asymptomatic and typically experience mild photophobia due to cystine crystals in the cornea observed accidently during a routine ocular examination. The ocular cystinosis is associated with different mutations in *CTNS* gene. Cysteamine therapy mostly corrects the organ abnormalities.

**Methods:**

This study was performed in collaboration with the department of ophthalmology of Farhat Hached Hospital. The Optical Coherence Tomography (OCT) of the cornea and retinal photography were used to search cystine crystals within the corneas and conjunctiva in eight Tunisian patients. Screening for the common 57-kb deletion was performed by standard multiplex PCR, followed by direct sequencing of the entire *CTNS* gene.

**Results:**

The studied patients were found to have cystine crystal limited anterior corneal stroma and the conjunctiva associated with retinal crystals accumulation. *CTNS* gene sequencing disclosed 7 mutations: three missense mutations (G308R, p.Q88K, and p.S139Y); one duplication (C.829dup), one framshift mutation (p.G258f), one splice site mutation (c.681 + 7delC) and a large deletion (20,327-bp deletion). Crystallographic structure analysis suggests that the novel mutation p.S139Y is buried in a first transmembrane helix closed to the lipid bilayer polar region, introducing a difference in hydrophobicity which could affect the hydrophobic interactions with the membrane lipids. The second novel mutation p.Q88K which is located in the lysosomal lumen close to the lipid membrane polar head region, introduced a basic amino acid in a region which tolerate only uncharged residue. The third missense mutation introduces a positive change in nonpolar tail region of the phospholipid bilayer membrane affecting the folding and stability of the protein in the lipid bilayer.

**Conclusions:**

Our data demonstrate that impaired transport of cystine out of lysosomes is the most common, which is obviously associated with the mutations of transmembrane domains of cystinosine resulting from a total loss of its activity.

## Background

Nephropathic cystinosis (MIM ≠ 219,800) is an autosomal recessive lysosomal storage disease due to the impaired transport of free cystine out of lysosomes. The disease is clinically classified into three different forms according to degree of renal disease severity: nephropathic cystinosis or renal Fonconi syndrome, intermediate cystinosis, and non-nephropathic or ocular cystinosis [1, 2].

Nephropathic cystinosis or renal Fonconi syndrome appears in the first year of life, with renal tubular involvement. The condition is caracterized by the accumulation of intralysosomal cystine within the cornea and conjunctiva in the eye. Other symptoms may occur in cystinosis patients, especially after adolescence; include muscle deterioration, diabetes, thyroid and nervous system problems. The intermediate cystinosis form presents the same sign as nephropathic cystinosis, but the occur symptoms at a later age. This form of disease typically becomes apparent in adolescence individuals. The kidney problems and corneal crystals are the most developed features of this disease form. The non-nephropathic or ocular cystinosis is caracterized by corneal cystine accumulation leading to photophobia, corneal erosions, and keratopathies [3] but usually without the development of kidney malfunction without the other signs and symptoms of cystinosis.

The three forms of cystinosis are caused by mutations in CTNS, which encodes lysosmal membran cystinosin. *CTNS* gene, (OMIM 606,272; GenBank NM_004937.2) is located on the short arm of chromosome 17 (13p) and contains 12 exons that are distributed across ~ 23 kb of genomic DNA. Cystinosin codes for a 367 amino-acid peptide, predicted to contain seven transmembrane domains (TM) topology and a tyrosine-based GYDQL lysosomal motif. The C-terminal tail is predicted to be oriented towards the cytosol and the highly glycosylated N-terminal region towards the lysosomal lumen [4, 5] and a novel conformational signal situated in the fifth inter-TM loop [6]. More than 140 mutations (www.hgmd.org. 2019) have been elucidated. Of note, the level of corneal crystal accumulation reflects on one hand the course and severity of the disease itself, and one the other hand the type of the *CTNS* gene mutation. There is inadequate information concerning the structure of cystinosin to infer the impact of this mutation upon functional domain of the protein. In the present study, *in silico* analysis on the functional and structural impact of the reported mutations helps to provide comprehensive insight into molecular mechanisms of cystinosin synthesis, function, and interaction with the lipid bilayer and to better understand the related clinical manifestations observed in eight Tunisian patients.

## Patients and methods

### Patients

This study was carried out on eight patients with cystinosis (P1 to P8) who have been diagnosed in the pediatric department of Sahloul Hospital Sousse, Tunisia. All investigated patients, except P8, were offsprings of consanguineous marriages between first or second cousins from different areas of Tunisia.

Family histories and main clinical data are reported in Table 1. All the affected children had healthy siblings.

**Table 1:** Clinical features and genetics profiles of patients with cystinosis

Family	Patients	origin	Consanguinity of parents/degree	Age of diagnosis (Yr/Mo)	Age (Yr/Mo)	Corneal crystal	Retinal deposits	Mutations	polymorphisms	Cystinosis subtypes	Reference
1	P1	Tunis		1 Yr		not analyzed	not analyzed	20,327 bp del	none	infantile	[7]
2	P2	Kondar; Sousse	second degree	1 Yr	10 Yr	Anterior stroma and posterior stroma in the periphery	No marked	p.G308R	rs161400_H_	infantile	[5, 7]
3	P3	Kairouan	second degree	1 Yr	8 Yr	Anterior stroma	diffuse	p.G258fs	none	infantile	[7, 8]
4	P4	Tunis	second degree	1 Yr	6 Yr	not analyzed	not analyzed	p.Q88K	rs161400_H_; rs156770260_h_; rrs1179007761_h_; rs459613_H_	infantile	This report
5	P5	Tunis		1 Yr		not analyzed	not analyzed	c.681 + 7delC	rs161400_H_; IVS10 + 34 C > A^a^_H_		This report
6	P6	Malloulich; Mahdia	second degree	3 Yr s	5 Yr	Anterior stroma	Diffuse with retinal atrophy	C .829dup	rs752919200; rs459613_H_; rs467277_H_, rs1450802529_h_	infantile	[9]
7	P7	Malloulich; Mahdia	second degree	1 Yr	8 Yr	Anterior stroma	Diffuse with retinal atrophy	C .829dup	Not analyzed	infantile	[9]
8	P8	Sekhira; Sfax	Unrelated parents	2 Yr	7 Yr 9 Mo	not analyzed	not analyzed	p.S139Y	rs5387504_H_; rs1323098109_h_; rs16400_H_; rs985161402_h_ rs1192394364, rs7469222242; rs459613, rs222753	infantile	This report

*Yr* Year, *Mo* Month, _*H*_ homozygous state, _*h*_ heterozygous state, ^a^ novel sequence variants

This study was approved by the Ethics Committee of the Sahloul Hospital (Sousse, Tunisia) and the families provided informed consent, prior to collecting blood samples. All procedures were in accordance with the ethical standards of the responsible committee on human experimentation (institutional and national) and with the Helsinki Declaration of 1975, as revised in 2000 and approved by the Ethics Committees of the respective Tunisian hospitals.

### Ocular surface analysis

All the studied patients were contacted to be present in an ophthalmologic consultation for examination. However, only six patients benefited from an ophthalmological examination as the other patients reside far away from the university hospital of Farhat Hached. The corneal crystals deposits screening is based on the slit lamp exam and anterior segment OCT which is a simple and non-invasive exam. It confirms the stromal localization of crystals.

Ocular symptoms were reported and noted. Corneal crystals were observed and located in their corneas. Anterior segment Optical Coherence Tomography (AS-OCT) was used to confirm cystine crystals accumulation within the corneas and conjunctiva of all studied patients. Retinal photography (RP) was carried out to show crystals deposits in the retina and to evaluate the retinal atrophy.

### Molecular analysis

Blood samples were collected from all patients and their parents.

Genomic DNA was isolated from leukocytes of patients with cystinosis according to the standard salting out procedure [10] .The DNA was used as a template for PCR amplification of the *CTNS* gene.

The PCR amplification of eight exons and intron-exon boundaries of the *CTNS* gene was carried out in 50 µL containing 50 ng genomic DNA, 0.2 mmol/L dNTPs, 0.4 pmol of each primer, 1.5 mmol/L MgCl_2_, 5% DMSO and 0.5 µL (5U/µL) Go TaqFlexy (Promega).

Amplification conditions included an initial 5 min denaturation step at 95 °C, followed by 35 cycles of denaturation at 95 °C for 35 s, annealing at 54 °C with the temperature ranging between 54 and 65 °C and extension for 1 min at 72 °C, followed by a final extension step for 7 min at 72 °C. The PCR products were purified and then utilized as templates for direct sequencing with the same PCR primers in both forward and reverse directions.

Sequencing was performed at the Laboratory of Biochemistry and Molecular Biology at the Béchir Hamza Children’s Hospital, Tunis as previously described [11].

### Molecular modeling

The computer-generated three-dimensional structure model of the human cystinosin was constructed by the protein homology modeling server SWISS-MODEL using the protein sequence retrieved from UniProt (UniProtKB id: O60931). The constructed tertiary structure was analyzed by DeepView Swiss-PdbViewer 4.1 and POV-Ray 3.6 software [7]. The transmembrane domains and the inter-transmembrane loops were identified and the previously identified mutations were localized in these domains. Further Crystallographic analysis and molecular dynamics simulation studies were performed to predict the effect of the novel missense mutations on protein functional and stability.

## Results

### Clinical finding

The dosage of intra-leukocyte cystine could not be carried out in our country; it was performed in the Metabolic Biochemistry Department, Hôpital Necker Enfants Malades, Paris, France. The diagnosis of our patients was based on the clinical, biological data of the cystinosis disease and was retained in view of the presence of De Toni Debré Fancon’s syndrome in the most studied patients (Table 1).

Patient with cystinosis appears normal at birth even though cystine accumulation already starts in utero. First symptoms occur in the most of the studied cohort at 18 months of the age with renal Fanconi syndrome, a dysfunction of the proximal tubule, polydipsia, polyuria, dehydration, proximal renal.

### Molecular finding

We analyzed eight patients belonging to eight different unrelated families from Tunisia exhibiting mild and severe phenotypes. PCR multiplex was performed, showing that none of the 8 studied patients carried the European 57-kb deletion in the *CTNS* gene. As result of DNA sequence analysis, 7 different mutations were identified including: p.Q88K, p.S139Y, p.G308R, c.681 + 7delc, C.829dup, p.G258fs and 20,327 bp deletion. In addition a large number of SNPs were identified.

#### Family 1/P1

The patient (P1) presented a positive family history for cystinosis. This infantile form of disease was homozygous for a large previously found deletion [8] and he exhibited cystine crystals limited to the anterior corneal stroma and conjunctiva.

#### Family 2/P2

The patient (P2) with the infantile form of disease, originating from the Center of Tunisia (Kondar) was homozygous for a previously missense mutation p.G308R [12]. This mutation was due of G transition to A at 922 genomic DNA position.

There was a family history; her brother had presented the same typical symptoms and died at the age of seven months. The prenatal diagnosis in this family showed that the fetus DNA was heterozygous for the c.922G > A (p.G308R) mutation.

**Fig. 1:**
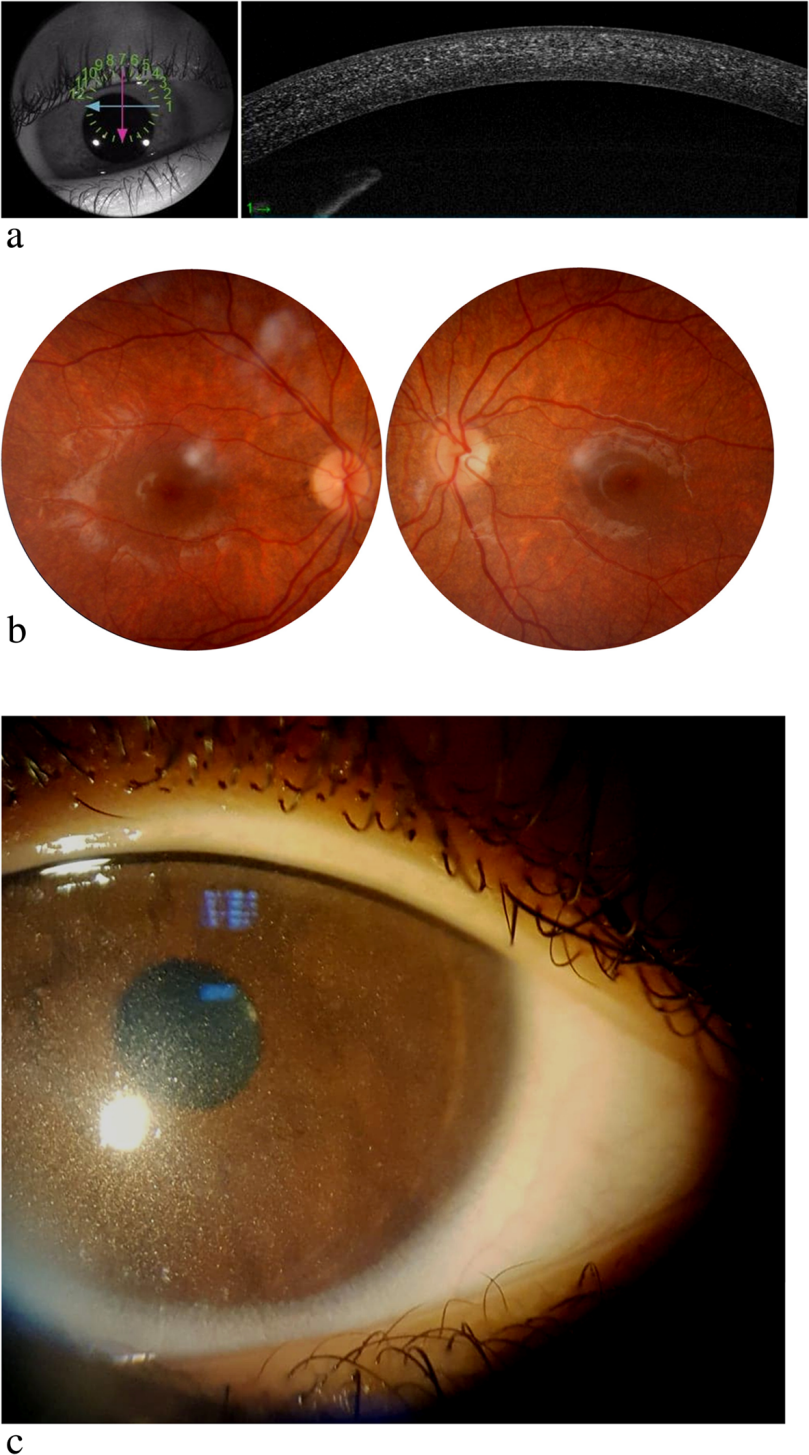
**a** AS-OCT of the patient P2 showing crystals corneal deposits in the anterior cornea at the center and in the totality of the stroma at the periphery. **b **Retinal photography of the patient P7 showing bilateral retinal crystals deposits andretinal pigment epithelium atrophy. **c **Corneal slit lamp examination of the patient P6 showing diffuse white and birefringent crystals in the cornea

 This patient was presented with moderate photophobia and tearing. He was under treatment (cysteamine eye drops) since few months. Best corrected visual acuity was 10/10. Slit lamp examination demonstrated anterior stromal corneal birefringent crystals accumulation confirmed by the AS-OCT that highlighted the crystals deposits (Fig. 1a). They were limited to the anterior corneal stroma in the center of the cornea but were affecting the posterior of the stroma in the periphery. RP did not allow highlight crystals deposits.

#### Family 3/P3

The patient (P3) with the infantile form of disease, was homozygous for a previously described frameshift c.771_793del (p.Gly258Serfs*30 ) mutation [12]. The studied patient was from a consanguineous marriage originated from the Center of Tunisia (Kairouan). The described frameshift mutation in exon 10 results in the deletion of ten nucleotides and produces premature termination of the CTNS glycopeptide.

This patient (P3) was presented with major photophobia and tearing. Visual acuity was not possible to evaluate. The Slit lamp examination demonstrated diffuse crystals accumulation in the cornea associated with cornea epithelium erosions. AS-OCT showed multiple crystals localized in the anterior stroma. The retina presented a particular localization of diffuse crystals deposits. Prenatal diagnosis on the third family showed that the fetus DNA had the same heterozygous mutation (c.771_793del (p.Gly258Serfs*30/Nl) as his parents.

#### Family 4/P4

The patient (P4), who presented the infantil form of cystinosis, was homozygous for the novel missense p.Q88k mutation. This mutation in exon 5 was a substitution of cytosine by adenine at 262 cDNA of *CTNS* gene.

#### Family 5/P5

The patient (P5) was homozygous for a novel splice site mutation at the donor site of intron 8. We have highlighted that the c.681 + 7delc splice site mutation activates a cryptic acceptor site (TAGgtatga) in intron 8, 86 nucleotides upstream (c. 662 + 86) with an HSF score of 86.88. This result suggests that the novel splice site mutation in intron 8 of the *CTNS* gene may cause either intron retention or cryptic splice site utilization.

#### Families 6 and 7/P6 and P7

The patients P6 and P7 with the infantile form of disease, were homozygous for a previously C.829dup mutation [13]. This duplication corresponds to a frameshift mutation (T277Nfs*19) starting at codon 277, leading eventually to a truncated protein at amino acid 296. The two unrelated patients (P6 and P7) were from a consanguineous marriage between first cousin living in the same region of Tunisia (Malloulich, Mahdia), which has been considered the focus of cystinosis.

The prenatal diagnosis in sixth family showed that the fetus DNA was normal for the C.829dup (T277Nfs*19) mutation.

Both patients P6 and P7 were presented with minor photophobia. Best corrected visual acuity was 8/10. Slit lamp examination demonstrated discrete deposits in the cornea. AS-OCTC showed limited anterior deposits. RP demonstrated diffuse crystals with marked retinal atrophy (Fig. 1b and c).

#### Family 8/P8

The patient (P8) with the infantile form of disease was found homoallelic for the novel p.S139Y missense mutation. This mutation was confirmed by monodirectional sequencing of the exon 7 of *CTNS* gene. The novel missense mutation was predicted pathogenic by Polyphen v.2 with a score of 1 (Available at http://genetics.bwh.harvard.edu/ggi/pph2). Analysis of pMut and SIFT yielded a probability of a deleterious mutation of 0.82 and 0.00, respectively. The studied patient was not from a consanguineous marriage, and she originated from the south of Tunisia (Mahres). The ophthalmologic test has not been done for this patient.

### Molecular modeling finding

**Fig. 2:**
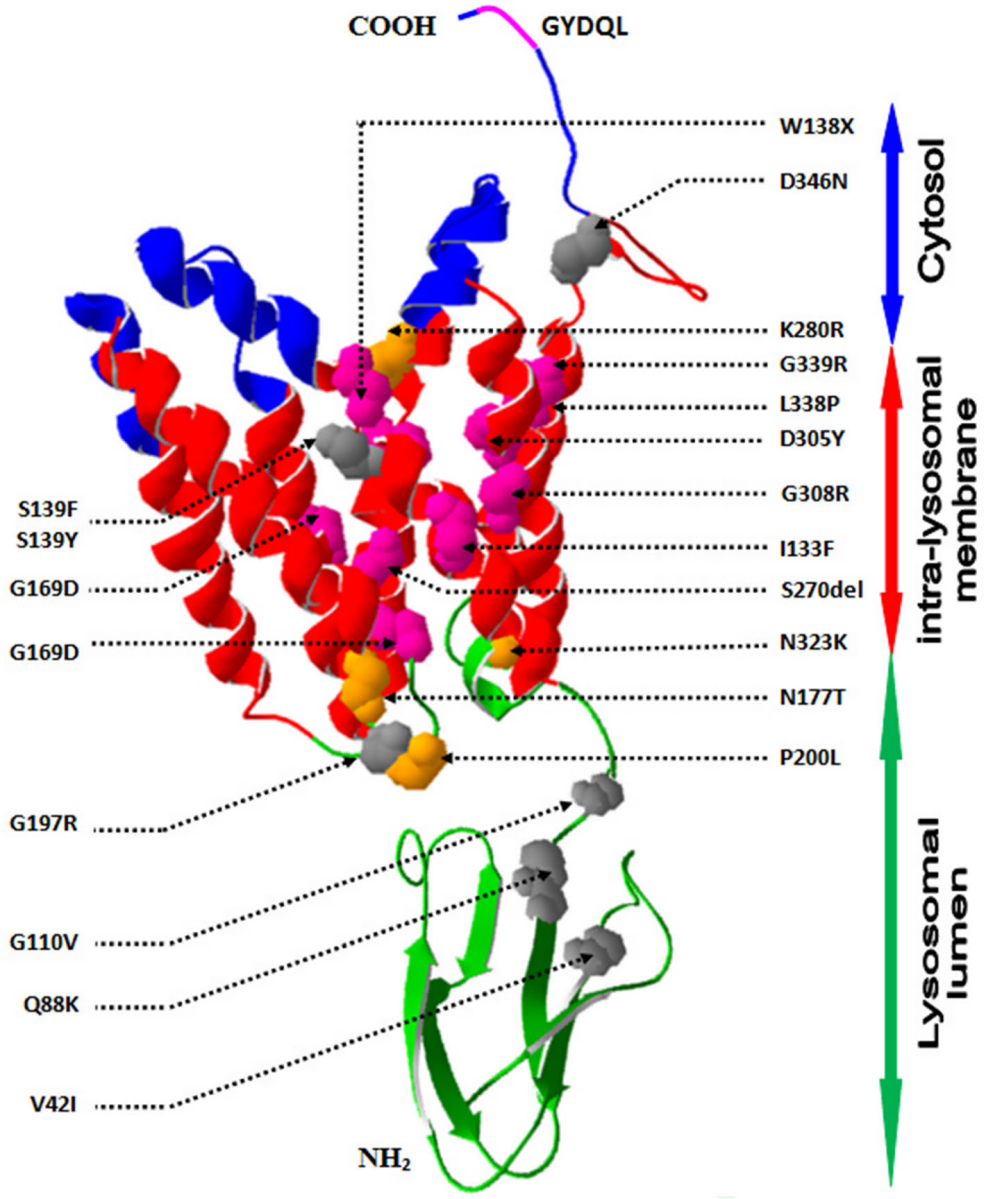
Crystallographic structure analysis of the computer-generated 3D structure model of the human Cystinosin showing its putative topology and the  reviously reported missense mutations. The localisation of the cystinosin regions in cytosol, intra-lysosomal membrane and lysosomal lumen are indicated by closed double arrow. The Transmembrane (TM) helices are colored in red in the ribbon structure, wherase the C-terminal tail and the N-terminal region are colored in blue and green, respectevely. Pink ribbon structure represents the lysosomal sorting signals: the GYDQL motif in the C-terminal tail. Previously described Infantile, Juvenile, Ocular and Atypical Cystinosis mutations are labeled and pointed by dotted arrow in the ribbon structure and colored in pink, orange, black and grey, respectevely. Molecules are oriented to best display all previously reported mutations The 3D structure file were generated from the protein homology modeling server SWISS-MODEL and the images were created using Swiss-PdbViewer 4.1.0 and POV-Ray 3.6 software

**Fig. 3:**
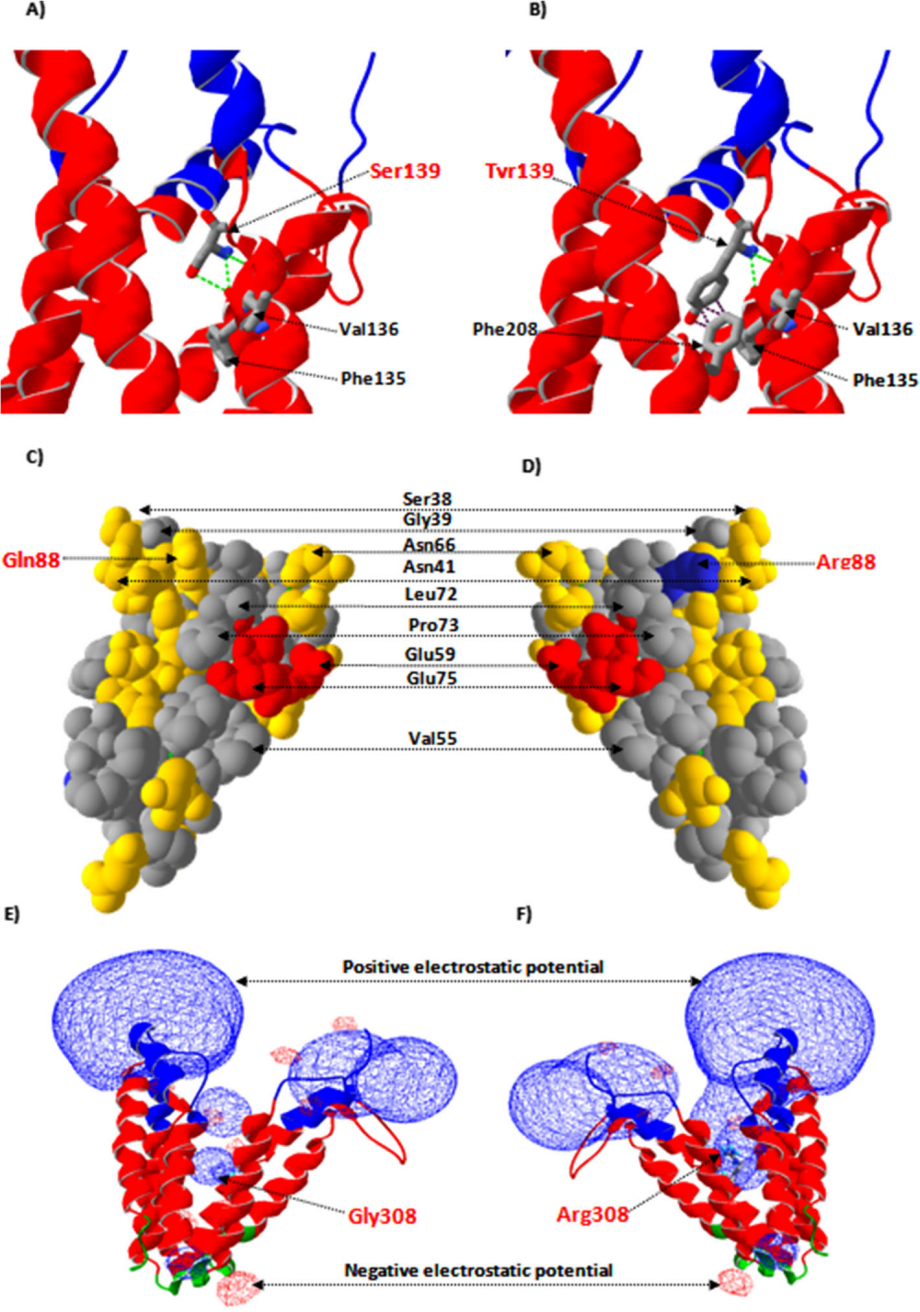
Modeling structure analysis of the reported three missense mutation Ser139Tyr (upper rows), Gln8Arg (Middel rows) and Gly308Arg (bottom rows). **a** the backbone of the wild type residue Ser139 form three hydrogen bonds with Phe135 and Val136 (shown as red dotted line), whereas (**b**) the mutated Tyr139 predicted to delete the third hydrogen bond between its side chain and the Val136 backbone and create a steric clash with the Phe208 (shown as black dotted line). **c** the Gln88 (polar amino acid (yellow)) is surrounded only by polar and nonpolar (grey) residues. Its substitution by Lys88 (**d**) introduced a basic amino acid in a region which tolerate only uncharged residue. For atom representation, acid residues are colored in red, basic in blue, polar in yellow and non-polar in grey. Electrostatic potentials were calculated using ionic strengths corresponding to 0 mM ion concentration and εP = 4 before (**e**) and after (bf) mutation (Gly308Arg) introduction. The mutated residue, buried in the transmembrane domain, introduce an important positive charge in the core of the proteine

 The Crystallographic structure analysis of the generated Cystinosin 3D structure model suggests that the novel mutation p.S139Y is buried in a first transmembrane helix closed to the lipid bilayer polar region. This mutation substitutes an amino acid with polar uncharged side chain into another with hydrophobic side chain. This difference in hydrophobicity can affect the hydrophobic interactions with the membrane lipids. On the other hand, further 3D structure analyzes demonstrate that the backbone of the wild type residue results in the formation of three hydrogen bonds with Phe135 and Val136 (Figs. 2 and 3). In contrast, its replacement with Tyr139 predicts to delete the third hydrogen bond between its side chain and the Val136 backbone, creating a steric clash with the Phe208 Benzylyl group buried in the third transmembrane helix. The repulsion between the mutant residue and neighboring residues could prevent the normal folding of the transmembrane domain and its transport in the lipid membrane.

Regarding the second novel mutation p.Q88K located in the N-terminal region, our crystallographic structure analysis demonstrates that the mutated amino acid is situated in the lysosomal lumen close to the lipid membrane polar head region. We notice that the most of the amino acid located in this analyzed region are and no polar residues, while this reported novel mutation introduced a basic amino acid K88 in a region which tolerate only uncharged residue.

The analysis of the electrostatic potential of the constructed model showed a positive charge distribution forming a characteristic electric field in the C-terminal tail which is situated in the cytosol. The missense mutation p.G308R, buried in the transmembrane domain, introduce an important positive charge in the nonpolar tail region of the phospholipid bilayer membrane. This mutation could affect the protein folding and stability or its interaction with the lipid bilayer.

The new physico-chemical properties introduced by the three reported mutations respectively could prevent the normal folding of the transmembrane domain and eventually may destabilize the conserved structure shared by most of the membrane transport protein. In addition the screening of polymorphisms in *CTNS* gene revealed the detection of 15 polymorphisms, at least (Table 1).

## Discussion

Cystinosis is a rare autosomal-recessive lysosomal storage disease caused by mutations in the *CTNS* gene that encodes the cystine transporter, cystinosin, which leads to lysosomal cystine accumulation. The most common form of cystinosis, the nephropathic or infantile type, is characterized by renal failure at the age of 10 years and other systemic complications and early corneal cystine crystal deposition. The Cornea is a common site for crystals accumulation. This is manifested by photophobia, the major ocular symptom in patients with cystinosis [14]. Deposits affect conjunctiva and retinal pigment epithelium (RPE) too. They can also be deposited in the iris, ciliary body, choroid and lens capsule.

This work was conducted as a straight continuation of studies carried out in other Tunisian cystinosis patients and their families [8].

Eight different Tunisian families with cystinosis were investigated in this study for both the molecular profile (CTNS mutations) and the ophthalmic examination. Consanguinity between first and second degree was reported in eight families. All of the probands initially presented with Fanconi syndrome. Cystinosis was suggested by the presence of crystals in the cornea in most of studied patients. Of note, the corneal crystal deposition starts a similar significant early age for all the studied patients, at the periphery of the cornea, with progression to the center and the posterior stroma [15], leading to corneal erosions, scarring, and neovascularization. They can also be deposited in the iris, ciliary body, choroid and lens capsule. However, in these Tunisian patients, the deposits affect only conjunctiva and retinal pigment epithelium (P1, P4, P6, P6 and P7) according to several studies [2].

In the current study, molecular analysis showed that none of the Tunisian patients had the 57-kb deletion. We have described four previously identified mutations: 20-kb deletion, p.G308R missense mutation, p.G258fs frameshift mutation; c.829 duplication; and three novel mutations including two missense mutations: p.G88K and p.S139Y and one splice site mutation IVS 8 + 7delC. All of the patients carried a homozygous CTNS mutation probably due to the high parental consanguinity rate.

### 20-kb deletion

This deletion was described for the first time in the homozygous state in a Tunisian patient who showed early onset cystinosis with severe features [8]. The functional test of the 20-kb deletion could not be further characterized in this study. The studied patient (P1) presented with the same clinical profile of her previously analyzed brother [8]. Additionally, the clinical profile of this patient was in agreement with several studies showing that the large deletion was always associated with the severe phenotype, resulting in cystinosin expression defect [12].

### p.G258fs mutation

The P3 patient was found homozygous for the described frameshift mutation which is located in exon 10, resulting in a small deletion of 23 nucleotides (c.771_793del), predicted to introduce premature termination of glycopeptides at 288 residue. This reported micro deletion is located in the sixth putative transmembrane domaine TM6. This mutation occurs at residues which are conserved between cystinosin and the transmembrane protein of yeast of *C. elegance* [9]. Furthermore, it has been noted that for the affected conserved residues or residues located in TMs of many other membrane protein, the transport activity is less tolerant [12], thus is similar with the G285, which is considered a conserved residue. Besides, this G285 mutation induces a loss cystin transport activity in the studied patient (P3) who presented crytals accumulation in the anterior stromal corneal. Besides, the cysteamine eye drops treatment during few months could at any age because direct placement of cysteamine solution on the cornea can dissolve cystine crystals in less than a year [2]. This mutation was identified for the first time in a patient presenting a classical infantile neuphropathic cystinosis [12] similar to the patient studied (P3).

### c.829dupA mutation

Both P6 and P7 patients with infantile nephropathic cystinosis were homozygous for the c.829dupA mutation. The c.829dupA is the most common mutation detected in Egyptian patients with infantile nephropathic cystinosis [16] but it has been reported only once in a heterozygous European patient [14].

The pathogenicity of this mutation caused by a single-base duplication and predicted to introduce a premature stop codon downstream that could also trigger nonsense-mediated decay. This is consistent with the severe phenotype observed in the unrelated patients (P6 and P7). The ophthalmologic examination showed diffuse crystals and retinal atrophy in the two patients. Furthermore, the lack of cystine transport activity of the CTNS- Glu227^*^ mutant may be associated with the frameshift mutation which is located at the 5th inter-transmembrane loop containing the PQ motif, required for H + and cystine co-transport [12].

### c.681 + 7delc mutation

The patient P5, with classical nephropathic cystinosis, was homozygosity for a novel splicing mutation in CTNS, c.681 + 7delc. Several studies showed that CTNS splicing mutations have been described in all three clinical variants of cystinosis: classic nephropathic cystinosis [12], the intermediate variant, and the ocular form. The c.681 + 7delc mutation in patient P5 within the ocular form was one of the eighth splicing mutations reported among nephropathic cystinosis patients. Splicing mutations generally occur early in the CTNS coding region and generate premature stop codons implicating the obliteration of the putative C-terminal tyrosine based lysosomal targeting signal.

### p.Q88K mutation

Patient P4 was homozygous for the novel missense mutation (p.Q88K) and showed the infantile cystinosis phenotype. Crystallographic analysis of the generated cystinosin 3D structure model demonstrates that most of the infantile cystinosis missense mutations are located in the transmembrane domains. In the literature, these mutations do not alter the lysosomal localization of cystinosin but abolish the cysteine transport [17]. In fact, EGFP-fused cystinosin constructs bearing 19 missense mutations associated with infantile cystinosis, were transiently expressed in HeLa cell to determine the subcellular localization and cystine transport activity of cystinosin [17]. The results suggest that the intralysosomal cystine accumulation is related to an inability to transport cystine rather than an expression defect. These mutations affect the normal folding of the cystinosin transmembrane domains in the phospholipid bilayer membrane resulting in infantile cystinosis phenotype.

On the other hand, missense mutations located in cystinosin regions oriented towards cytosol or lysosomal lumen region are usually associated with the juvenile or ocular phenotype. HeLa cell expression of these mutations show a low level of cystine transport compared to those associated to infantile patient cystinosis [17]. In the present study, the novel mutation Gln88Lys which is buried in the lysosomal lumen, close to the lipid membrane region, represents an exception since it is associated with infantile cystinosis. The charge introduced by the mutated residue could disturb the interaction between the cystinosin N-terminal region and phospholipid bilayer polar head region, leading to impaired or limited transport activity which contrasts with the severe phenotype. About the second novel missense mutation Ser139Tyr situated in the first transmembrane helix, crystallographic analysis demonstrated that this mutation affects the hydrophobic interactions with the lipid bilayer polar region. Interestingly, another mutation has been previously reported in the same amino acid residue Ser139Phe and was associated with an atypical cystinosis phenotype. The carrier of this mutation, classified as atypical due to a late onset of renal Fanconi syndrome revealed only at the age of 3 years [12], compared to P8 in this study (Table 1). The physico-chemical properties of each mutated residue could explain the difference in phenotype expression between these cases.

### p.G308R mutation

Both the P2 patient and her sister (who died at 1 year and six months old) showed severe phenotype with growth retardation, renal tubular fonconi syndrome, polyuria, corneal cystine crystals and elevated leukocyte cystine levels. Mutational screening revealed a missense mutation p.G308R affecting a highly conserved amino acid residue among vertebrates, since it is essential for preserving a particular neutral charge in the sixth transmembrane domain of cystinosin. Based on these chemical properties, the positive charge introduced by the mutated residue Arg308 could affect the interaction between the transmembrane domain and the nonpolar tail region of the phospholipid bilayer membrane. Interestingly, COS cells expressing of cystinosin bearing the p.Gly308Arg mutation greatly affect the ability of the protein to transport cystine at the plasma membrane [5]. In the literature, this mutation was detected in homozygous and compound heterozygous states in German and Swiss patients respectively and also associated with the severe infantile nephropathic phenotype.

The screening of polymorphisms in *CTNS* gene revealed that there are at least 13 polymorphisms detected and was associated with the severe infantile nephropatic form of disease, (Table 1). Here, we describe one novel polymorphisms in intron 10 within patient P5. The polymorphism was in the homozygous status.

It is noteworthy that the unclassified sequence change, p.I260T (refSNP ID: rs161400) co-segregates with all the identified mutations in the studied patients (P2, P4, P5, and P8) who presented the infantile form of cystinosis; as described in the literature [17]. Thus, to understand the origin of a mutation, it is very useful to identify whether a certain recurrent mutation is associated with the same haplotype.

## Conclusions

In this and our previous study, the finding data demonstrate that the mutational spectrum of the Tunisian patients is particular and different from patients in other countries, probably due to on one hand, the heterogeneous origins of the population and on the other hand due to the still high proportion of marriages between first cousins.

A special emphasis like in vivo confocal microscopy and ultrasound biomicroscopy should be placed on a multidisciplinary approach to diagnosis in order to achieve the best clinical outcome of cystinosis.

## Data Availability

The datasets used and analyzed during the current study are available from the corresponding author upon request. The mutations of the CTNS gene were submitted to ClinVar database (https://www.ncbi.nlm.nih.gov/clinvar/) under accession number : SCV001573761, SCV001573194 and.

## Declarations

The study was approved by the ethics committees for scientific research of the Sahloul Hospital Sousse, Tunisia; no reference number was issued.

All procedures were in accordance with the ethical standards of the responsible committee on human experimentation (institutional and national) and with the Helsinki Declaration of 1975, as revised in 2000 and approved by the Ethics Committees of the respective Tunisian hospitals.
